# Review of Recent Advances in Intelligent and Antibacterial Packaging for Meat Quality and Safety

**DOI:** 10.3390/foods14071157

**Published:** 2025-03-26

**Authors:** Junjun Zhang, Jianing Zhang, Lidan Zhang, Zhou Qin, Tianxing Wang

**Affiliations:** Agricultural Product Processing and Storage Lab, School of Food and Biological Engineering, Jiangsu University, Zhenjiang 212013, China; junjun_5457@ujs.edu.cn (J.Z.); zjn821224@163.com (J.Z.); 19710510382@163.com (L.Z.); 19599960979@163.com (Z.Q.)

**Keywords:** active packaging, intelligent packaging, meat freshness, meat preservation, meat safety

## Abstract

Intelligent and antimicrobial packaging technologies are transforming meat preservation by enhancing food safety, enabling real-time quality monitoring, and extending shelf life. This review critically examines advancements in intelligent systems, including radio frequency identification (RFID), gas sensors, time-temperature indicators (TTIs), and colorimetric indicators for continuous freshness assessment. A key focus is natural compound-based chromogenic indicators, which establish visual spoilage detection via distinct color transitions. Concurrently, antimicrobial systems integrating inorganic compounds, organic bioactive agents, and natural antimicrobials effectively inhibit microbial growth. Strategic incorporation of these agents into polymeric matrices enhances meat safety, supported by standardized evaluation protocols for regulatory compliance and quality assurance. Future research should prioritize optimizing sensitivity, cost-efficiency, and sustainability, alongside developing biodegradable materials to balance food safety with reduced environmental impact, advancing sustainable food supply chains.

## 1. Introduction

With the rapid development of the global economy, people’s consumption levels have been steadily rising. Meat products have become an indispensable part of modern diets. As a primary source of animal protein and fat in daily nutrition, meat demonstrates substantial consumption and market demand [1]. According to the United Nations Food and Agriculture Organization (FAO), the global average per capita meat intake reached 35.5 kg in 2022 [2]. Furthermore, the OECD-FAO Agricultural Outlook projected that worldwide meat consumption would increase to 129 million tons during the 2022–2031 period. Within the composition of meat consumption, fresh meat accounts for 70–80% of total consumption. Meat proteins are biologically valuable and rich in essential amino acids. Due to its high nutritional value and flavor, meat has garnered increasing attention. Most meats contain 16.5% to 20% protein. In addition to protein, meat is rich in fatty acids (such as linoleic acid, linolenic acid, and oleic acid), micronutrients (including various minerals), and vitamins (such as thiamine, riboflavin, niacin, and other B-complex vitamins) [3]. However, compared to other food categories, meat is highly perishable and requires proper processing methods to extend its shelf life. The degradation of meat primarily involves lipid and protein oxidation, which is accelerated by factors such as moisture, air, light, and processing temperatures. The phospholipids and triglycerides in meat, particularly those containing unsaturated fatty acids, are especially susceptible to oxidation [4,5]. Oxidation induces a range of undesirable alterations in color, appearance, flavor, and texture, consequently diminishing the nutritional value of the substance [6,7]. In meat stored under certain conditions, microflora activity can lead to the formation of harmful metabolites such as toxic amines, ketones, or sulfur derivatives, which are rapidly decomposed [8]. These components directly lead to the degradation of meat’s nutritional properties and pose significant risks to human health. Therefore, it is imperative to implement scientifically validated and effective technologies for monitoring meat freshness and preventing food safety incidents. Consequently, food packaging serves as a crucial protective barrier for meat products, safeguarding them against physical damage (e.g., extrusion and collision) and environmental factors (e.g., sunlight exposure and chemical contamination) [9,10,11]. The packaging materials function as effective barriers against air, water vapor, and other gaseous substances, thereby maintaining meat quality. Over the past decade, extensive research has been conducted on advanced packaging solutions, particularly focusing on intelligent and active packaging systems for meat products [12,13,14,15]. As an innovative advancement in meat preservation, intelligent packaging systems integrate real-time monitoring capabilities throughout the food supply chain, enabling immediate transmission of meat quality parameters to stakeholders including producers, retailers, and consumers [16,17]. This technology facilitates the visualization and quantification of critical indicators such as pH levels [18], CO_2_ concentration [19], and O_2_ content [20], thereby significantly mitigating potential food safety risks. Intelligent packaging represents a technological breakthrough that synergistically combines multiple functional elements from diverse scientific disciplines, including physics, chemistry, and biology, through advanced multi-integrated systems [15]. This integration endows packaging materials with intelligent characteristics, enabling dynamic quality monitoring and real-time feedback to regulatory bodies. The comprehensive functionality of intelligent packaging encompasses five core aspects: quality monitoring, product protection, data recording, information communication, and preservation enhancement [21]. The integration of intelligent packaging with advanced network technologies has created a promising frontier in the meat industry, representing a significant growth opportunity. Current research on intelligent packaging primarily focuses on three distinct technological categories: information-based systems, sensor-embedded solutions, and indicator technologies [22]. Information-based systems typically utilize RFID and QR codes, while sensor-embedded solutions often incorporate biosensors and gas sensors. Indicator technologies commonly employ color-changing dyes responsive to pH or microbial activity.

In addition, microbial invasion is one of the primary causes of meat spoilage and deterioration. Contamination by various microorganisms, such as fungi and bacteria, leads to food waste, which has significant implications for both the environment and human health [23]. Therefore, ensuring meat quality and extending its shelf life are crucial. Antibacterial packaging not only helps reduce physical damage but also minimizes the impact of microorganisms on meat [24]. In response to these preservation challenges, high-performance antimicrobial packaging has emerged as an innovative solution. This advanced packaging technology extends meat shelf life and maintains product quality through the controlled modification of the internal environment. The antimicrobial agents are uniformly distributed within the packaging matrix, with their efficacy enhanced by controlled-release mechanisms that facilitate migration through the packaging materials to the food surface [25]. These agents can reach any space within the packaging, which can kill microorganisms on the surface of the food and inhibit the growth of microorganisms in the food packaging space [26].

**Figure 1:**
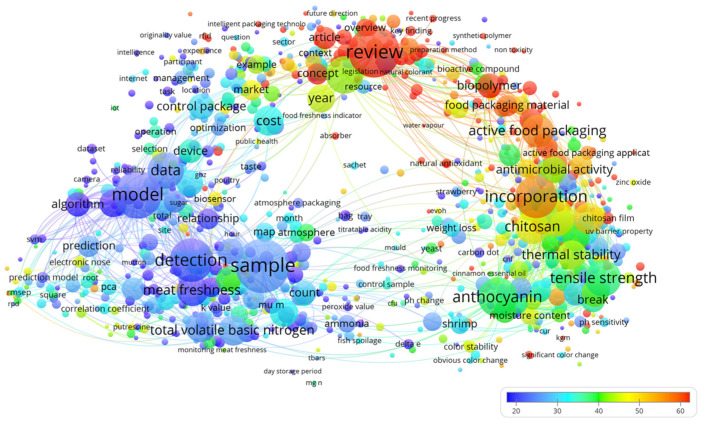
The keywords co-occurrence network diagram of meat packaging.

In recent years, escalating demands for food safety and quality management have positioned active packaging, intelligent packaging, and meat safety as critical research frontiers [27]. To systematically map interdisciplinary trends and technological convergence, this review employs a bibliometric analysis of keyword co-occurrence patterns, supported by VOS viewer-1.6.18 software. Figure 1 presents the keyword co-occurrence network, revealing two dominant clusters, active packaging and intelligent packaging, distinguished by their large node sizes (indicating high citation frequency) and dense interconnections (reflecting technological convergence). Notably, these clusters exhibit functional complementarity, suggesting synergistic potential in practical applications. A distinct sub cluster focusing on meat freshness assessment emerges, characterized by key terms such as TVB-N, detection methods, and sample analysis. This reflects the field’s reliance on chemical analytics and intelligent monitoring systems to evaluate meat quality. The color-coded cluster distribution further demonstrates interdisciplinary research diversity, with mechanical properties, thermal stability, and antimicrobial activity forming a cohesive research axis. This underscores a dual paradigm: optimizing functional packaging performance while advancing novel material development for food packaging applications. The analysis identifies three persistent research trajectories: (1) the integration of active and intelligent packaging systems for enhanced synergy; (2) advanced intelligent systems enabling real-time monitoring; and (3) innovative freshness assessment methodologies to improve accuracy [28].

While numerous review articles have examined the territory of intelligent and active packaging films in food quality and safety, a comprehensive analysis of functional categories specific to meat quality remains unexplored. This review aims to address this research gap by providing a detailed examination of advanced packaging systems, with emphasis on intelligent monitoring and antimicrobial agents in meat preservation applications.

## 2. Intelligent Packaging for Monitoring Meat Quality

Intelligent packaging has emerged as a transformative technology in food preservation, serving as a critical interface for monitoring and responding to environmental interactions between packaged products and their surroundings. This advanced packaging technology provides consumers with essential product information and early quality indicators, facilitating informed decision-making processes. Over the past decade, diverse intelligent packaging systems, incorporating various operational principles, have been successfully implemented across the meat industry [29,30].

### 2.1. Radio Frequency Identification (RFID)

As a pivotal information technology in food packaging, radio frequency identification (RFID) systems digitally encode comprehensive product information within microchips, including product name, ingredients, functional properties, origin, shelf life, weight, pricing, and usage instructions [31]. This technology enables consumers to access essential food information through non-contact, wireless radio wave transmission, offering significant advantages over traditional identification methods. While barcodes and QR codes remain prevalent patterns for encoding basic product details in machine-readable formats, RFID technology eliminates the need for physical connection devices and enables automated product identification through wireless communication between food labels and interrogators [32]. The implementation of RFID systems facilitates comprehensive online monitoring of meat safety throughout the entire supply chain, from production to consumption. The compact dimensions of RFID tags permit flexible placement on food packaging or even subcutaneous implantation in animals [33]. A complete RFID system comprises three core components: electronic tags, readers, and database infrastructure. The tags function as information carriers, storing critical data for product identification and traceability. Readers serve as communication interfaces, transmitting interrogation signals and receiving decoded data responses from tags. The backend database system, operating either independently or integrated within enterprise information systems, processes and analyzes RFID data to support advanced applications and inform decision-making processes.

The application of RFID technology in meat packaging offers several advantages, enhancing the efficiency, safety, and traceability of products throughout the supply chain. One of the most critical applications of RFID in meat packaging is improving traceability. An RFID system comprises a specific chip for monitoring the meat quality of the supply chain from transport to storage period. Recent studies demonstrate that RFID systems can acquire comprehensive online data from tags without requiring physical scanning devices, offering superior data collection capabilities compared to traditional methods [33]. RFID-enabled meat packaging facilitates real-time inventory tracking and stock level monitoring, ensuring optimal product freshness. Each RFID tag stores detailed product information, including origin, processing dates, and complete temperature histories, enabling full supply chain visibility from production to retail. This enhanced traceability significantly improves food safety protocols and enables the rapid response to potential contamination incidents or product recalls, as evidenced in recent smart packaging research [31]. Advanced RFID implementations integrate multiple sensor technologies for comprehensive quality monitoring. Temperature-sensitive RFID tags were successfully deployed to maintain optimal storage conditions throughout the supply chain, with automated alert systems triggering corrective actions when deviations occur [34]. These integrated solutions not only reduce food waste but also promote sustainable business practices by decreasing the environmental impact of meat production. RFID technology has emerged as a pivotal tool for real-time meat quality tracking. Recent advancements combined RFID tags with temperature, humidity, and ammonia sensors to dynamically track microbial growth of pork freshness quickly and accurately [35]. For instance, Nando et al. [36] developed a passive RF to monitor pork spoilage during storage, achieving 96.944% accuracy in freshness prediction through machine learning algorithms. Similarly, Abounasr et al. [37] designed an RFID-enabled antenna-based sensor, which wirelessly transmitted beef spoilage data via the sensor, demonstrating high correlation (R^2^ =0.984) with simulated data. These examples underscore RFID’s versatility in bridging digital traceability and biochemical quality assessment.

While RFID systems represent a transformative technology for supply chain visualization and waste reduction in modern meat packaging, their implementation faces notable challenges. The relatively high production costs associated with RFID technology may limit its accessibility for small-scale operations and price-sensitive market segments, potentially restricting widespread adoption among ordinary consumers and businesses.

### 2.2. Gas Sensors

In meat product packaging systems, the dynamic composition of internal atmosphere gases serves as a critical indicator of product quality and storage conditions. Gas sensors, typically implemented as compact tag-based devices, are strategically integrated into packaging films to monitor and detect quality-related changes in meat products [38,39]. Li et al. developed a thin film coated with dye-sensitized TiO_2_ on interdigitated electrodes [40]. When the sensing film interacts with volatile alkaline nitrogenous compounds, particularly putrescine and other biogenic amines released during pork spoilage, the titanium dioxide (TiO_2_) semiconductor material undergoes an electron transfer process. This interaction results in the capture of electrons from the volatile compounds, subsequently modifying the electrical resistance characteristics. This resistance change typically occurs within 2–5 min of gas exposure, with sensitivity thresholds reaching 1–5 ppm for putrescine detection, demonstrating rapid response characteristics suitable for real-time meat quality monitoring.

Through precise measurement of the electrical signal variations, the system enables quantitative detection of volatile alkaline gas concentrations emitted from meat products throughout critical stages of the supply chain, including transportation, processing, and storage [41]. This electrochemical sensing mechanism provides a reliable method for pork freshness assessment [42]. In a significant advancement of this technology, Al Obaidi et al. developed an intelligent gas-impermeable film incorporating CO_2_-sensitive chromogenic dyes for poultry freshness monitoring. Their research demonstrated distinct visual color transitions from blue to green, correlating with progressive storage duration and quality degradation [19]. In a complementary study focusing on skinless chicken breast freshness, researchers developed an innovative food freshness indicator system. This system established a direct correlation between colorimetric variations and CO_2_ concentration levels. To enhance the visual detection capability, the study employed two distinct chemical dye mixtures: one combining bromothymol blue and methyl red, and another incorporating bromothymol blue, bromocresol green, and phenol red. These formulations significantly improved both the range and sensitivity of color transitions in response to spoilage metabolites, demonstrating dynamic freshness indication through visible color changes from green to red [43]. However, such colorimetric sensors exhibit certain limitations, including sample specificity and the requirement for relatively high CO_2_ concentrations typically associated with advanced spoilage stages. During meat storage, microbial decomposition of proteins predominantly generates volatile ammonia compounds, making ammonia detection a more prevalent approach for freshness assessment. Shi et al. [44] developed an advanced silk-based composite sensor integrating titanium dioxide (TiO_2_) and polyaniline, demonstrating enhanced performance in pork freshness evaluation. The results indicated that the fabricated micro-sensor exhibited a response value of 0.82 to ammonia gas (100 ppm) with a response time of 10 s. Additionally, the linear discriminant analysis (LDA) model used to evaluate pork freshness showed a recognition rate of 90.73% for the calibration set and 86.38% for the prediction set, demonstrating a strong linear correlation. Enisa [45] et al. studied two novel biosensors based on putrescine oxidase and diamine oxidase for monitoring meat freshness. The results revealed that the biosensors had a quantitative linear range for putrescine between 1 and 2 mg/L, with detection limits of 0.8 mg/L and 1.3 mg/L, respectively. By analyzing the quality changes in meat over an 8-day storage period, the feasibility of using biosensors to detect putrescine was confirmed, with results comparable to high-performance liquid chromatography analysis. However, the aforementioned sensors are susceptible to signal drift and significant interference from background signals, which limits their application in meat storage processes. In another study, Valdez et al. proposed an ammonia and biogenic amines gas sensor based on polydiacetylene nanofibers for colorimetric detection of spoilage in various meat (chicken, beef, fish, and pork). The actual meat freshness monitoring tests confirmed a visible color change from blue to red, indicating food freshness [46]. A summary of different types of gas sensors in meat quality applications is provided in Table 1.

### 2.3. TTIs

Time-temperature indicators (TTIs) have emerged as essential tools for comprehensive quality monitoring in meat supply chains, delivering critical real-time data that influences multiple aspects of food management, including quality assurance, consumer confidence, and regulatory adherence [53].

Time-temperature indicators (TTIs) represent a critical class of smart packaging solutions designed to systematically track and document the cumulative thermal history of perishable meat products. These devices operate as continuous monitoring systems, capturing temperature fluctuations across the entire supply chain continuum from production to retail, thereby serving as biochemical chronometers for quality assurance [54]. The TTI responds to temperature fluctuations by undergoing either a reversible or irreversible color change, allowing consumers, retailers, and suppliers to assess whether the meat has been stored under appropriate conditions during transportation, storage, and display. These indicators are often integrated into packaging materials, such as labels, and stickers, or directly into the packaging film. For meat products, TTIs are particularly useful in detecting temperature abuse, which is a key factor in spoilage and foodborne illnesses [55]. By providing real-time data on temperature fluctuations, TTIs help ensure that meat products remain safe and of high quality, thereby enhancing quality control and minimizing the risk of microbial contamination [56]. A common application involves color-changing labels that gradually shift from green to red when the meat is exposed to temperatures above a specified threshold. This real-time monitoring prevents spoilage by ensuring that meat is stored within safe temperature conditions [57]. Antonia et al. [58] developed an OnVu™ TTI kinetics system for chilled meat and predicted the discoloration process. Giannoglou et al. [59] integrated a TTI system for fish freshness, where enzymatic hydrolysis of lipid substrates generates pH changes, triggering color transitions correlated with microbial growth. In addition to maintaining product quality, TTIs offer several key benefits, including extending shelf life, reducing food waste, and improving food safety by alerting consumers and retailers to potential temperature abuse that could lead to bacterial growth or pathogen contamination [59].

However, despite their many benefits, TTIs have certain limitations. They primarily monitor temperature and do not account for other factors that affect meat quality, such as humidity, oxygen levels, or the presence of harmful microorganisms, all of which can contribute to spoilage. Furthermore, while TTIs effectively indicate historical temperature abuse, many existing systems employ irreversible mechanisms that prevent resetting or reuse. This characteristic particularly restricts their application in scenarios requiring continuous temperature surveillance throughout the product’s entire shelf life. Moreover, integrating TTIs into meat packaging can increase production costs, making it difficult for small-scale producers or low-cost meat products to adopt the technology. However, the future of TTIs in meat packaging holds great promise, particularly with advancements in integrating TTIs with other smart technologies, such as gas sensors and RFID systems, to create a more comprehensive quality monitoring system.

### 2.4. Colorimetric Indicator Packaging

Colorimetric indicator packaging is a promising technology that incorporates color-changing indicators into packaging materials to detect the quality and freshness of meat products in real-time. This smart packaging works by using chemical or biological dyes that respond to specific changes in the meat’s environment, such as pH shifts [60,61], microbial growth [46,62], or oxidation [63], which are common signs of spoilage. When these indicators detect such changes, they undergo a color shift, visually signaling the degree of freshness or spoilage. The advantage of colorimetric indicators lies in their simplicity and non-invasiveness, offering consumers, retailers, and logistics providers an easy-to-interpret visual cue without the need for additional tools or complex measurements. For example, Bambang Kuswandi et al. developed a polyaniline-based packaging film capable of monitoring the freshness of fish. Their results demonstrated a positive correlation between the total bacterial count and the color change in the polyaniline film and in the fish during storage, allowing spoilage to be indirectly assessed by observing the color of the film [64]. The indicators used in these studies are synthetic chemical dyes, which pose certain toxicity risks and potential threats when applied to meat packaging. Consequently, there is a need for non-toxic, natural, and safe substances to serve as indicators in intelligent packaging. In fact, natural pigments show different structural forms with different colors at different pH values [65]. These natural pigments alter color in response to changes in pH of the meat, offering a straightforward visual information for meat freshness [66]. The natural plant pigments mainly contain anthocyanins, curcumin, alizarin, and betaine naphthoquinone. As detailed in Table 2, recent applications of natural pigments in intelligent meat packaging systems demonstrate their viability for monitoring product freshness and quality.

The meat packaging industry currently faces significant challenges, requiring intelligent packaging solutions that can monitor meat quality in real-time to meet the growing consumer demand for high-quality meat products. As meat spoils, it undergoes a series of changes that result in the production of various biogenic amines, such as ammonia, trimethylamine, and dimethylamine. These biogenic amines, collectively referred to as total volatile basic nitrogen (TVB-N), serve as key physicochemical indicators in national standards for assessing meat freshness. The microbial decomposition of proteins in meat leads to an increase in TVB-N levels, gradually shifting the packaging environment to an alkaline state. This phenomenon provides the scientific foundation for developing colorimetric indicators that detect meat freshness by sensing pH changes within the packaging.

#### 2.4.1. The Application of Colorimetric Indicators Based on Anthocyanins

**Figure 2:**
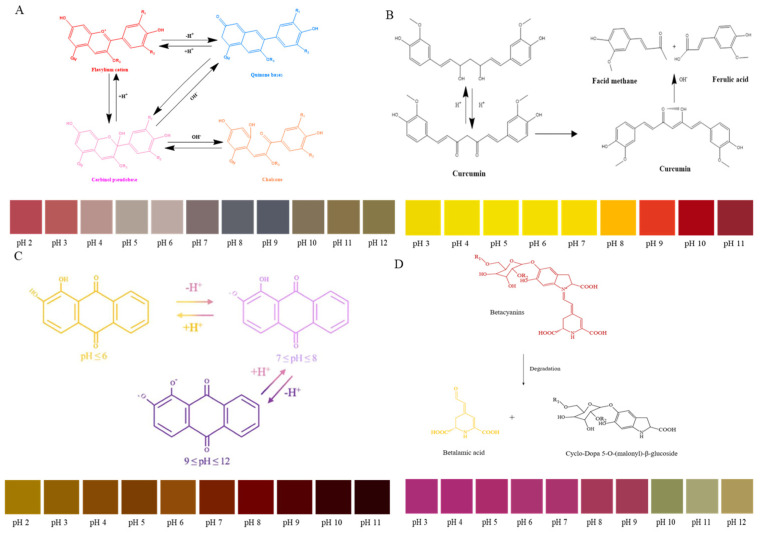
Structure transformation and color changes in natural pigments ((**A**): anthocyanin; (**B**): curcumin; (**C**): alizarin; (**D**): betaine).

The increasing level of TVB-N caused by meat spoilage can gradually transform into an alkaline environment of the package [67]. The pH indicator can serve as a freshness marker to assess meat quality. Anthocyanins, with their strong potential as natural pH indicators, offer a reliable and rapid tool for real-time detection of meat quality [68]. As can been seen in Figure 2, the structure transformation and color changes in the natural pigments (include anthocyanin, curcumin, alizarin, betaine) are obviously different in pH solutions [69]. The development of colorimetric indicators utilizing natural pigments has emerged as a promising approach for meat freshness monitoring [70,71]. Among these, anthocyanin-based indicators have gained prominence due to their natural origin and distinctive color-changing properties. These plant-derived pigments, typically extracted from sources such as red cabbage and various berries, offer an eco-friendly alternative to synthetic indicators. Recent advancements in biodegradable materials and intelligent packaging technologies have further enhanced their application potential [17,65,72]. A notable innovation by Zhang et al. [68] demonstrated the successful integration of purple Clitoria ternatea anthocyanins with biodegradable polymers (agar and starch) to create an intelligent packaging film for shrimp freshness monitoring. This system exhibited a characteristic color transition from blue to yellow corresponding to increasing storage duration, providing a reliable visual indication of meat quality. Consequently, anthocyanin-based intelligent packaging systems have attracted substantial research interest, particularly for their combination of natural origin, biodegradability, and effective sensing capabilities. As noted in Table 2, increasing research attention has been directed towards the use of anthocyanins in developing pH indicators for freshness monitoring of various meat products in recent years [73,74]. For example, anthocyanin extracted from the blue butterfly pea was developed into packaging film for chicken breast freshness monitoring. Visible color changes in the indicator were observed as storage time increased, confirming its efficiency and sensitivity in real-time monitoring of chicken breast freshness [75]. Recently, some researchers developed a new type of pH-sensitive film by incorporating bacterial cellulose and anthocyanins from perilla leaves. It was found that the freshness of shrimp could be determined by observing distinct color changes in the film (i.e., fresh (purple), sub-fresh (gray), and spoiled (yellow)) as the storage time of the shrimp sample increased [76]. In a comprehensive study, Kan [77] compared the effectiveness of adding anthocyanins from 14 different plants to polyvinyl alcohol/starch films for monitoring the freshness of pork and shrimp samples. Freshness-indicating films with good traceability offer customers real-time information about food quality and safety. This helps minimize the distribution of unsafe or poor-quality meat, while also enhancing the sustainability of the food supply.

**Table 2:** The application of indicator films based on natural pigments for meat packaging.

Source	Meat	Temp.	Time	Color Changes	Ref
Barberry	lamb	25 °C	72 h	Red to yellow	[74]
*Clitoria ternatea*	chicken	25 °C	48 h	Blue to green	[75]
Perilla	pork	25 °C	48 h	Red to yellow	[78]
*Echium Amoenum*	shrimp	4 °C	4 d	Purple to yellow	[76]
Roselle	pork	25 °C	36 h	Red to yellow	[16]
Blueberry	lamb	10 °C	72 h	Pink to colorless	[79]
Mulberry	pork	4 °C	6 d	Red to blue	[80]
Curcumin	shrimp	4 °C	36 h	Yellow to orange	[81]
Curcumin	Beef	4 °C	4 d	Yellow to brown	[82]
Curcumin	chicken	4 °C	5 d	Yellow to red	[83]
Alizarin	rainbow trout	4 °C	4 d	Yellow to red	[84]
Alizarin	fish	4 °C	6 d	Yellow to purple	[85]
Alizarin	beef	4 °C	3 d	Yellow to pink	[86]
Red pitaya peel	shrimp	20 °C	24 h	Red to yellow	[87]
Cactus pear	shrimp	4 °C	5 d	Pink to yellow	[88]

#### 2.4.2. The Application of Colorimetric Indicators Based on Curcumin

Due to its pH sensitivity, curcumin can serve as an indicator material in intelligent packaging to monitor food spoilage [81]. As meat produces alkaline gases during spoilage, the pH value in the packaging environment changes, causing the color of curcumin to shift. This color change provides spoilage information to both producers and consumers [89]. In recent years, there has been increasing research on the development of intelligent packaging using curcumin for the rapid, non-destructive, and real-time evaluation of meat freshness [90,91]. For example, curcumin was used as a pH indicator to prepare hydrogels for monitoring shrimp freshness. During the deterioration of the shrimp, the color of the curcumin film transformed from yellow to orange-red, which could be easily observed by the human eye. The experiment confirmed that curcumin could be effectively applied as an indicator to monitor the shrimp freshness [81].

In addition, some researchers have used low-density polyethylene and curcumin as raw materials to prepare highly hydrophobic indicator films, which can accurately reflect freshness changes in beef and silver carp under high humidity conditions. As the TVB-N levels in the sample increase, the hydrophobic film changes from yellow to brown [82]. Recently, Yildiz and colleagues [83] used curcumin, chitosan, and polyethylene oxide to prepare electrospun films for monitoring chicken freshness. The fiber film exhibited high biocompatibility, non-toxic degradability, and high sensitivity to volatile ammonia. In a storage environment at 4 °C, the film effectively indicated changes in the freshness of chicken breast meat, with the color transitioning from bright yellow to red.

#### 2.4.3. The Application of Colorimetric Indicators Based on Alizarin

The color change range of alizarin aligns with the pH shifts associated with meat spoilage, making it an effective colorimetric indicator for intelligent films. For example, a novelty pH-sensitive sensor was developed by incorporating alizarin into starch–cellulose paper for rainbow trout filets freshness monitoring. Significant color changes were observed, which corresponded closely with the TVB-N values of the fish, providing a reliable indicator for identifying the onset of spoilage in refrigerated fish filets [84]. Agaei et al. [85] also prepared a nanofiber indicator film by incorporating alizarin into zein via electrospinning technology for fish freshness. During the storage process, the color of the nanofiber film also showed yellow to purple on the 6th day, and the film turned magenta once the fish had spoiled. Additionally, some researchers developed color-changing indicator films using alizarin and two different types of cellulose (CMC and CNF). These indicator films exhibited UV-light screening properties and enhanced thermal stability, demonstrating their potential for monitoring meat freshness [92]. Recently, a film containing alizarin into PLA and chitosan was prepared. The composite film features an antibacterial zone at the edge and a freshness indicator zone at the center. The results demonstrated that the composite film possesses strong UV-barrier and antibacterial properties. During the storage of the chicken breast, the film exhibited significant color changes, making it a useful indicator of food freshness [93]. In our previous study, alizarin was grafted onto ZIF-8 to improve color stability. The colorimetric film provided an early signal of beef freshness, with the color changing from yellow to pink at 4 °C [86].

#### 2.4.4. The Application of Colorimetric Indicators Based on Betaines

Betaines have great potential as natural pH dyes for real-time, in situ detection of food freshness through color changes. For example, a study developed an active and intelligent indicator film incorporated with betalains-rich red pitaya (*Hylocereus polyrhizus*) peel extract to monitor shrimp freshness. The results showed that the color of the indicator film changed significantly in an ammonia-rich environment. Moreover, as storage time increased, the color shifted from red to yellow, demonstrating the effectiveness and sensitivity of real-time shrimp freshness monitoring [87]. Another study developed an ammonia-sensitive film by combining polyvinyl alcohol, quaternary ammonium chitosan, and cactus pear extract. Real-time, in situ detection of shrimp freshness was achieved by observing significant color changes in the film during the freshness indication process. The color shifted from pink to orange and finally to yellow. Additionally, the film exhibited active properties due to its antioxidant and antimicrobial characteristics [88]. The researcher also developed intelligent films using betaine-rich extracts from red dragon fruit pulp, cactus fruit, red beet, amaranth, and red amaranth leaves to monitor shrimp quality in subsequent studies. The betaine composition in different plants was analyzed. As TVB-N levels increased, the color of the film gradually shifted from purple/red to yellow. The results indicated that red pitaya extract was the most suitable for monitoring shrimp freshness with obvious color changes [94]. Recently, a systematic review of natural pigment-based intelligent films found that betacyanins were more sensitive than naphthoquinone, curcumin, anthocyanin, and alizarin [66]. However, research on betaine in the field of intelligent films is relatively limited compared to other pigments, presenting a significant opportunity for future development.

Intelligent packaging offers excellent traceability, providing customers with real-time information about the quality and microbial contamination of meat. This helps minimize the distribution of unsafe meat and enhances the sustainability of the food supply chain. Recent studies have shown that natural pigment indicator films have a promising future and will significantly contribute to the development of intelligent packaging systems. However, while intelligent packaging can provide real-time information on meat freshness, it cannot prevent spoilage. Therefore, there is a growing demand for antibacterial packaging with preservative effects to prolong the shelf life of meat products, requiring intervention throughout the meat’s shelf life.

## 3. Antibacterial Packaging for Meat Preservation

Meat antimicrobial packaging is a critical tool in addressing the challenges of food safety, spoilage, and quality assurance. Nowadays, antimicrobial packaging has become a hot topic in the meat packaging industry [95,96]. This innovative approach integrates active ingredients into packaging materials to inhibit the growth of microorganisms, thereby extending shelf life and enhancing meat safety. Spoilage microorganisms and pathogenic bacteria not only compromise the quality of meat but also pose serious health risks to consumers, contributing to foodborne illnesses and outbreaks [97]. Active meat packaging is primarily based on natural, edible biopolymer substances (proteins, polysaccharides, cellulose, and their derivatives), blended with small amounts of food additives (such as emulsifiers, plasticizers, antioxidants, food colorants, flavors, and antimicrobial agents) [98]. These films form a porous network structure through various intermolecular interactions [99]. Furthermore, antimicrobial packaging films serving as carriers for active molecules must meet stringent safety and functional requirements. These materials must maintain complete non-toxicity and either remain flavor-neutral or impart pleasant aromas when in direct contact with meat surfaces. Additionally, optimal barrier properties are essential to minimize moisture loss and maintain meat quality. The development of edible antibacterial packaging materials represents a particularly promising advancement, as these innovative solutions can be safely consumed along with the packaged food products, offering significant potential for sustainable food industry applications. Building on the intelligent packaging principles discussed earlier, the following sections critically analyze antimicrobial technologies, exploring their various aspects and applications. The analysis begins by deconstructing the sources and mechanisms of active ingredients commonly used in meat packaging. Next, different types of antimicrobial packaging products are discussed. Finally, the focus shifts to the growing field of edible antimicrobial packaging, highlighting its potential to extend shelf life, maintain quality, and align with sustainability goals in the meat industry.

### 3.1. Antibacterial Ingredients in Meat Packaging: Source and Mechanisms

The effectiveness of antibacterial packaging relies on active ingredients that inhibit microbial growth, thereby extending the shelf life of meat products. These ingredients can be categorized into inorganic, organic, and natural antibacterial agents, each with unique mechanisms suitable for meat packaging applications.

#### 3.1.1. Inorganic Antibacterial Ingredients and Their Mechanisms

Inorganic antibacterial agents are known for their stability and effectiveness under different environmental conditions, making them suitable for meat packaging. They primarily include metal ion-based and photocatalytic antibacterial agents. Metal ion antibacterial agents (such as Ag^+^, Zn^2+^, and Cu^2+^) exert their antibacterial effects through contact killing, catalytic killing, and the inhibition of ATP synthase activity [100]. Photocatalytic antibacterial agents primarily utilize inorganic materials like Nano-TiO_2_ and metal Na, which generate hydroxyl radicals (OH-) through photoelectron transition, leading to oxidative sterilization.

The use of metal-based antibacterial materials in food packaging can inhibit the growth of pathogenic microorganisms, enhance sensory characteristics, and extend the shelf life of food [101]. Metals like Ag, Cu, Zn, Fe, Mn, Al, Mg, Sr, Co, Ce, Ni, Sn, Zr, Cd, and Ba all exhibit antimicrobial properties; however, due to considerations of cost, safety, and efficiency, Ag, Cu, and Zn are the most commonly used in meat packaging applications. The antimicrobial mechanisms of different metal ions are similar. For example, silver and silver nanoparticles primarily function through the release of Ag+ ions, which interact with bacterial proteins. These interactions include forming S-Ag bonds with thiol groups in cysteine and other compounds, disrupting electron transport chains, and interfering with DNA replication and synthesis [102]. However, single-metal antimicrobial materials have inherent limitations. Ag+ ions are prone to oxidation, which reduces their antibacterial effectiveness, while the color of copper ions can interfere with the appearance of antimicrobial materials [102]. To enhance the antimicrobial properties of existing metal-based meat packaging and overcome the limitations of the single-metal agents, some studies have focused on combining and doping multiple active ingredients. This approach improves the physicochemical properties of the materials, creating synergistic effects that enhance antimicrobial performance. However, concerns over metal ion migration and potential toxicity must be addressed to ensure their safe application in direct-contact meat packaging [103]. Therefore, it is crucial to assess the migration of antimicrobial components to food surfaces. Although many studies have evaluated the migration of metal antimicrobial agents, a comprehensive toxicity evaluation system for these agents in meat packaging has not yet been established.

Photocatalytic reactions can also be used to inactivate bacteria and viruses by oxidizing essential biochemical components. These reactions were employed to deactivate pathogens such as *E. coli*, *hepatitis B virus*, and *influenza virus* [104]. When electron acceptors or donors are adsorbed onto the semiconductor surface, photoelectrons will react with electron acceptors, while holes will react with electron donors, leading to redox reactions that effectively kill bacteria on the packaging surface [105]. For example, Nano-TiO_2_ particles are used to create active food coatings that inhibit microbial proliferation through photocatalytic oxidation of unsaturated phospholipids. The antibacterial mechanisms of Nano-TiO_2_ involve the generation of highly oxidative substances (e.g., superoxide anions) under UV irradiation, which oxidize bacterial cellular polysaccharides, proteins, lipids, and nucleic acids [106,107]. The application of photocatalytic materials in meat packaging enhances microbial safety, but their effectiveness varies depending on bacterial resistance, with Gram-negative bacteria often showing higher resistance than Gram-positive bacteria.

#### 3.1.2. Organic Antibacterial Ingredients and Their Mechanisms

Organic antibacterial agents are widely used in meat packaging due to their rapid sterilization capability and compatibility with packaging materials. Organic acids such as lactic acid, sorbic acid, and tartaric acid lower pH levels and disrupt bacterial metabolism, making them effective for meat preservation [108]. Among these, organic acid antimicrobial agents are the most widely studied and used in meat packaging [109]. Organic acids have two main mechanisms of action for inhibition and sterilization [110]. One mechanism is lowering the pH of the bacterial environment, while the other involves non-dissociated organic acids, which can pass through the cell membrane via free diffusion. Once inside the cell, these acids dissociate into carboxyl ions and H+ ions. Carboxyl ions inhibit DNA replication and protein synthesis and disrupt bacterial cell membrane function, while the dissociated H+ ions further lower the pH inside the bacterial cell.

#### 3.1.3. Natural Antibacterial Ingredients and Their Mechanisms

Natural antibacterial ingredients are derived from plants, animals, and microorganisms, providing a safer and more eco-friendly alternative for meat packaging applications. However, these ingredients generally exhibit poor heat resistance and have relatively short efficacy periods [111]. Based on their sources, these antibacterial ingredients can be categorized into plant-derived, animal-derived, and microorganism-derived types.

Ancient Egyptians used plant juices for their antiseptic properties, while ancient Chinese civilizations employed various herbs to treat diseases [112]. Research has shown that the antibacterial ingredients found in plant metabolites include terpenoids and their derivatives, alkaloids, saponins, steroids, lignans, amino acids, and antimicrobial peptides [113,114]. The antibacterial mechanisms of plant-derived ingredients involve disrupting or degrading the cell walls of pathogens, damaging the cytoplasmic membrane and proteins, and causing cytoplasmic coagulation [115]. Essential oils (EOs), a typical example of plant extracts, are considered secondary metabolites of plants. They consist of aromatic, aliphatic, and terpenoid compounds, as well as minor nitrogen and sulfur compounds [116]. EOs are incorporated into matrix materials by coating or placing them in antimicrobial packaging bags. However, the challenges include poor water solubility, volatility, and strong odors, which limit their application [117]. The controlled-release rate of EOs is critical for effective antimicrobial action and avoiding rapid depletion [118]. Encapsulation or loading EOs in controlled-release packaging materials is a growing research focus to reduce odor and enhance stability, solubility, and bioactivity [119].

Animal-derived antibacterial ingredients include chitosan, amino acids, high-molecular-weight sugars, and natural peptides, with chitosan and natural peptides being the most widely studied [120,121,122,123]. Chitosan is particularly well suited for antibacterial packaging due to its film-forming ability, antibacterial properties, and biodegradability [124,125]. The electrostatic attraction between the positively charged chitosan chains and the negatively charged bacterial cell walls leads to cell adsorption and ultimately causes cell death [126]. However, chitosan’s poor mechanical properties limit its direct application in meat packaging, requiring blending with other polymers to enhance performance.

Microorganism-derived antibacterial ingredients include bacteriophages, probiotics, and bacteriocins [127,128,129]. Among these, bacteriocins are gaining popularity due to their heat and acid resistance and are produced ribosomally by bacteria during metabolism [130]. Nisin, a widely used bacteriocin produced by *Lactococcus lactis*, has an antimicrobial mechanism that involves electrostatic interactions between its positively charged structure and the negatively charged bacterial cell membrane [131,132]. This interaction causes leakage of cellular contents, such as DNA, leading to bacterial death. Nisin is particularly effective against Gram-positive bacteria, including *Micrococcus luteus*, *S. aureus*, and *Bacillus cereus*, and is commonly used in combination with polymer-based packaging materials to improve meat safety [133].

#### 3.1.4. The Evaluation Methods of Antibacterial Packaging

Various methods have been employed to evaluate the antimicrobial efficacy of meat packaging. The inhibition zone test is the simplest method [134]. In this test, the antimicrobial film is placed on a bacterial culture medium, where the antimicrobial agents in the film kill or inhibit microbial growth, forming a clear or inhibited zone. The results are expressed as the diameter of the inhibition zone. While straightforward, this method can be influenced by numerous experimental variables, such as film size and properties, microbial species, agar medium, temperature, and incubation time. Another method is the plate count, where the film is placed in a microbial bouillon culture medium [135]. The sample solution is then spread onto an agar plate, and the colonies are counted, providing microbial counts and measuring the logarithmic phase of bacteria. Other methods focus on determining the film’s ability to release antimicrobial components, providing information on the release rate or the quantity of antimicrobial compounds released over time. These evaluation techniques help develop safer and more efficient antibacterial packaging for meat products.

### 3.2. The Applications of Active Packaging Films on Meat Preservation

Antimicrobial packaging films have emerged as a key technology in meat preservation, offering a practical and efficient solution to microbial contamination and spoilage [136]. These advanced films serve dual protective functions: preventing microbial contamination of meat products while simultaneously providing effective barriers against oxygen transmission and moisture migration [137]. With excellent antibacterial ability, a variety of antimicrobial packaging has been applied to extend the storage time of meat and seafood products. Meat products are a vital component of modern diets worldwide [138]. Given the high perishability of meat, microbial proliferation can rapidly alter its pH, texture, color, and safety, leading to deterioration and economic loss. Antimicrobial packaging films have been extensively developed to control microbial activity, extending the shelf life of fresh, processed, and frozen meat products [139]. Several studies have demonstrated that edible antimicrobial films ensure the safety of meat products by inhibiting microbial growth [140,141]. The primary function of antimicrobial films in meat preservation lies in the controlled-release of antimicrobial agents [142]. The release kinetics of these agents are predominantly governed by the affinity of essential components within the film matrix rather than chemical bonding interactions [143]. The extended release of antimicrobial ingredients from the packaging film on the food surface can be more effective than incorporating the antimicrobial substances directly into the food [144,145]. Given the diverse physicochemical properties of different meat products (e.g., fresh beef, poultry, and seafood), the selection of an appropriate antimicrobial film requires careful consideration of film composition, antimicrobial agent compatibility, and meat-specific storage conditions.

To optimize the effectiveness of antimicrobial packaging in meat applications, researchers have explored different film preparation techniques, including the direct immersion of films in antimicrobial solutions, coating technologies, and nanocomposite-based approaches. These strategies enhance the stability, controlled-release, and antimicrobial efficacy of packaging materials used for raw, processed, and ready-to-eat meat products. In recent years, an increasing number of researchers have focused on developing advanced active antimicrobial films specifically designed for meat packaging applications. Marzlan et al. [146] investigated the remarkable potential of turmeric inflorescence-based antibacterial films in extending the shelf life of refrigerated chicken. The experimental results demonstrated that chicken samples treated with the antibacterial film exhibited significantly lower microbial counts and reduced thiobarbituric acid reactive substance (TBARS) values after six days of refrigeration. This innovative approach effectively enhanced product quality and extended shelf life while maintaining the original sensory characteristics of the chicken throughout the refrigeration period. Similarly, Roy and colleagues [147] demonstrated the efficacy of clove essential oil-incorporated packaging films in extending the shelf life of stored pork products. Göksen [148] developed active packaging films by incorporating polyvinyl alcohol with rosemary essential oil for chicken breast preservation, which demonstrated significant effectiveness in reducing total microbial counts during storage. In a separate investigation, researchers successfully integrated anthocyanins extracted from purple sweet potato into a composite matrix of polyvinyl alcohol and agar, creating an innovative antimicrobial film with pH-responsive properties. The distinct colorimetric changes exhibited by the antimicrobial indicator pads enabled real-time monitoring of meat spoilage, effectively extending the product’s shelf life by a minimum of 24 h [149]. However, active components in packaging systems are susceptible to dissolution and migration phenomena. Consequently, antimicrobial films intended for meat products, meat additives, meat contact materials, or meat packaging materials must strictly adhere to regulatory standards established by national authorities, such as the European Parliament and Council Directive No. 95/2/EC and FDA regulations (2006). Notably, the FDA has granted Generally Recognized as Safe (GRAS) status to certain antimicrobial films approved for human consumption.

## 4. Future Trends

Intelligent packaging systems, incorporating real-time sensors (e.g., pH, CO_2_, O_2_), are revolutionizing meat supply chains by enabling data-driven interventions that enhance transparency and reduce waste. Concurrently, antimicrobial packaging technologies—utilizing controlled-release agents such as essential oils, organic acids, and nanoparticles—extend shelf life but face critical limitations. A key challenge lies in the premature release of active compounds during early storage phases when microbial activity is negligible, resulting in depleted efficacy during later spoilage stages. This mismatch between release kinetics and microbial growth dynamics highlights the need for synchronized, demand-driven delivery mechanisms to optimize preservation outcomes [150].

To address this challenge, controlled-release packaging systems offer a promising solution by ensuring the gradual and targeted release of antimicrobial agents in response to microbial proliferation. An optimal release profile would feature minimal discharge during the early storage phase, followed by a substantial release as microbial activity increases, thereby maximizing preservation efficacy. The design of such systems must carefully balance release kinetics with microbial growth patterns. If the antimicrobial release rate surpasses microbial growth rates, active compounds may be depleted prematurely, compromising long-term efficacy. Consequently, optimized controlled-release packaging systems offer a scientifically validated approach to address these challenges in the meat industry. Future advancements in controlled-release packaging will likely focus on the integration of stimuli-responsive materials (e.g., pH-sensitive or enzyme-activated systems), as these innovative packaging solutions can achieve “on-demand” release mechanisms, ensuring sustained antimicrobial protection during critical spoilage phases. Future advancements will critically depend on the integration of artificial intelligence-driven predictive models to optimize release profiles and enable customized packaging solutions for diverse meat products. While the existing literature reviews predominantly focus on generic applications of active and intelligent packaging, this work uniquely synthesizes cutting-edge developments in smart sensing technologies, precision antimicrobial delivery systems, and engineered controlled-release mechanisms specifically tailored for meat preservation. This comprehensive analysis provides a strategic roadmap for next-generation packaging solutions that effectively balance food safety, environmental sustainability, and consumer confidence.

Looking ahead, research in antimicrobial active packaging will primarily concentrate on investigating the interactions between antimicrobial components and food safety parameters, thereby establishing edible antimicrobial active packaging as a dominant industry trend. Furthermore, antimicrobial active packaging materials are anticipated to catalyze transformative changes across the food industry, revolutionizing preservation methods and product quality standards. The synergistic integration of intelligent and antimicrobial packaging technologies represents a promising paradigm for contemporary meat preservation. Future research directions should prioritize the optimization of these systems to achieve enhanced sensitivity, cost-efficiency, and environmental sustainability. Furthermore, the development of eco-friendly, biodegradable packaging materials is crucial for minimizing environmental impact while maintaining food safety standards. These continuous technological advancements in packaging systems are expected to significantly contribute to reducing food waste, bolstering consumer confidence, and fostering a more sustainable global food supply chain. The widespread adoption of these innovations faces significant challenges, primarily due to the elevated production costs associated with novel antimicrobial ingredients. The industrial-scale implementation of edible packaging necessitates extensive research efforts to enhance product quality and optimize production cost-efficiency. Therefore, limiting experimental approaches exclusively to the perspective of meat packaging represents a constrained research paradigm. Instead, it necessitates an interdisciplinary framework that integrates diverse fields such as microbiology, materials engineering, and food science, requiring collaborative efforts among experts from various industries to advance the research and development of antimicrobial packaging systems. Furthermore, the design and development of antimicrobial packaging must rigorously comply with both domestic and international food safety regulations and standards. The implementation process should strictly adhere to established review and validation mechanisms, ultimately evolving into a comprehensive industrial chain encompassing the entire research, development, and commercialization of antimicrobial packaging technologies.

## 5. Conclusions

In recent years, intelligent and antimicrobial packaging technologies have emerged as innovative solutions for real-time meat quality monitoring and shelf life extension. Intelligent packaging systems, encompassing radio frequency identification (RFID), gas sensors, time-temperature indicators (TTIs), and colorimetric indicator packaging, have demonstrated substantial potential in ensuring meat safety through continuous quality assessment. Particularly, colorimetric indicators utilizing anthocyanins, curcumin, alizarin, and betaines provide a simple yet effective visual detection method for meat freshness based on pH and environmental changes.

Simultaneously, antimicrobial packaging has proven essential in suppressing microbial proliferation and maintaining meat quality. Extensive research has investigated various antimicrobial agents, including inorganic compounds, organic substances, and natural ingredients, for their efficacy against spoilage and pathogenic microorganisms. The incorporation of these antimicrobial compounds into packaging matrices has significantly enhanced meat safety and extended shelf life, with rigorous evaluation methods ensuring both effectiveness and regulatory compliance.

Recent advancements in intelligent and antimicrobial packaging technologies have demonstrated significant potential in enhancing the shelf life, safety, and quality parameters of meat products. Ranging from sophisticated time-temperature indicators to advanced antimicrobial packaging systems, these innovations are fundamentally transforming contemporary food preservation practices. Nevertheless, substantial challenges persist in terms of regulatory compliance, cost-effectiveness, and consumer adoption, which must be systematically addressed to facilitate widespread implementation. Future research endeavors should prioritize the development of sustainable, eco-friendly materials and the optimization of precision controlled-release mechanisms, thereby enhancing the efficacy, accessibility, and commercial viability of these innovative packaging solutions.

## Figures and Tables

**Table 1:** The application of gas indicators in meat packaging.

Gas Type	Mechanism	Sample	Response	Ref
CO_2_	bromothymol blue (BTB)	poultry	from blue to green	[19]
H_2_S	myoglobin	broiler	from brown to green	[47]
aldehyde	polydimethylsiloxane	chicken	phase differences	[48]
H_2_S	copper acetate	poultry	colorimetric response	[49]
ammonia	TiO_2_ and polyaniline	pork	0.82 (100 ppm)	[44]
CO_2_	mixed-dye	chicken	from green to yellow	[3]
H_2_S	AgNPs	carp	yellow to colorless	[50]
gaseous amines	red-cabbage	beef, chicken, shrimp whiting	pink to green-blue	[51]
O_2_ CO_2_	fluorescence	mutton, chicken, beef, pork, and fish	5000 and 7 ppm for CO_2_ and O_2_	[20]
volatile amine	organic semiconductor	fish	200–300 ppb	[52]

Notes: ‘Gas type’: This refers specifically to the target gas(es) inside the packaging environment; ’Mechanism’ describes the detection principle or working mechanism of the sensor.

## Data Availability

No new data were created or analyzed in this study. Data sharing is not applicable to this article.
